# Burnout in podiatrists associated with individual characteristics, workplace and job satisfaction: A national survey

**DOI:** 10.1002/jfa2.12003

**Published:** 2024-04-03

**Authors:** Daniel R. Bonanno, Anna Couch, Terry Haines, Hylton B. Menz, Belinda G. O'Sullivan, Cylie M. Williams

**Affiliations:** ^1^ School of Allied Health, Human Services and Sport La Trobe University Melbourne Victoria Australia; ^2^ Allied Health Peninsula Health Frankston Victoria Australia; ^3^ School of Primary and Allied Health Care Monash University Frankston Victoria Australia; ^4^ School of Rural Health Monash University Bendigo Victoria Australia

**Keywords:** burnout, healthcare, podiatry, workforce

## Abstract

**Background:**

Burnout is highly prevalent among health practitioners. It negatively impacts job performance, patient care, career retention and psychological well‐being. This study aimed to identify factors associated with burnout among Australian podiatrists.

**Methods:**

Data were collected from registered podiatrists via four online surveys administered annually from 2017 to 2020 as part of the Podiatrists in Australia: Investigating Graduate Employment (PAIGE) study. Information was collected about work history, job preferences, personal characteristics, health, personality, life experiences and risk‐taking behaviours. Multiple logistic regression analyses were used to determine if (i) individual characteristics, (ii) workplace factors and (iii) job satisfaction measures were associated with burnout (based on the abbreviated Maslach Burnout Inventory).

**Results:**

A total of 848 responses were included, with 268 podiatrists (31.6%) experiencing burnout. Participants experiencing burnout were slightly younger, more recent to practice, had poorer health, greater mental distress, lower scores for resilience, extraversion, agreeableness, conscientiousness, emotional stability and openness to experiences. They were less likely to have financial and clinical risk‐taking behaviour and more likely to have career risk‐taking behaviour. Prediction accuracy of these individual characteristic variables for burnout was 72.4%. Participants experiencing burnout were also more likely to work in private practice, have more work locations, work more hours, more direct patient hours, see more patients, have shorter consultation times, more likely to bulk bill chronic disease management plans, have less access to sick leave and professional development and be more likely to intend to leave patient care and the profession within 5 years than participants not experiencing burnout. Prediction accuracy of these workplace‐related variables for burnout was 67.1%. Participants experiencing burnout were less satisfied with their job. Prediction accuracy of these variables for burnout was 78.8%.

**Conclusions:**

Many of the factors associated with burnout in Australian podiatrists are modifiable, providing opportunities to implement targeted prevention strategies. The strength of association of these factors indicates high potential for strategies to be successful.

## 1 BACKGROUND

Burnout, a psychological syndrome resulting from chronic workplace stress [1, 2], is highly prevalent among health practitioners [3, 4, 5, 6]. A survey published in 2004 revealed that 25% and 30% of newly qualified podiatrists in the United Kingdom and Australia experienced burnout, respectively [4]. Burnout remains a common problem affecting podiatrists. A recent survey focusing on Australian podiatrists found that 35% of respondents experienced burnout, which was associated with an intention to leave the profession [5].

Individuals experiencing burnout may feel overwhelming exhaustion, mental detachment or cynicism towards their work and feelings of reduced professional efficacy [1]. In addition to the personal effects on health practitioners, burnout can contribute to adverse patient satisfaction, lower‐quality patient care and an increase in medical errors [7, 8, 9]. This poses a risk to patient safety, resulting not only in compromised care but also disruption to collaborative healthcare efforts [10].

Identification of factors (both individual characteristics and factors related to their work environment) associated with health practitioners experiencing burnout could facilitate identification and targeting of preventative strategies [6]. Previous research identified that a lack of professional status and geographic and professional isolation is associated with burnout among recently qualified podiatrists [4]. However, as over two decades have passed since this research was published and burnout remains a prevalent problem, there is a need for a contemporary assessment of this issue with the podiatry profession. As burnout is recognised as a multi‐level phenomenon [11, 12], it is best to examine a diverse range of factors encompassing both individual and work‐related elements. Therefore, an updated exploration of a comprehensive set of factors is needed for creating predictive models capable of identifying podiatrists most at risk of experiencing burnout.

The primary aim of this study was to determine if (i) individual characteristics, (ii) workplace factors and (iii) job satisfaction measures were associated with burnout among Australian podiatrists.

## 2 METHODS

### 2.1 Study design

This cross‐sectional study used workforce participation data collected from registered Australian podiatrists between 2017 and 2020, using information from the Podiatrists in Australia: Investigating Graduate Employment (PAIGE) study [13]. Data were collected via four waves of online surveys, with each wave remaining open for approximately 6 months each year. Ethical approval was provided by the Human Research Ethics Committee of Monash University (19959). All participants provided informed consent prior to survey completion. The findings from this study have been reported according to the Checklist for Reporting Results of Internet E‐Surveys (CHERRIES) [14].

### 2.2 Participants and setting

Australian podiatrists were invited to participate in all four waves of surveys. The first (2017) and second (2018) waves were limited to podiatrists registered to practice in Victoria (*n* = 1440) [5]. All podiatrists registered to practice in Australia (*n* = 5429) were eligible to participate in the third (2019) and fourth (2020) waves (Version 1.2) [15]. The survey was promoted at Australian podiatry conferences, social media (Facebook, Twitter/X, LinkedIn and Instagram) and through targeted emails from peak bodies such as the Australian Podiatry Association and Australasian College of Podiatric Surgeons. Participants were also directly invited via email to complete each wave of the survey if they had completed a survey in the previous year.

### 2.3 Survey design

All survey data were collected online using Qualtrics® software (Qualtrics, Seattle, WA, USA). Participants were asked to identify past responses which dictated question logic. When participants completed the survey for the first time (in any year) they were asked questions relating to job satisfaction, location, training and questions about their podiatry practice such as setting, hours of work and hours spent providing direct patient care. Where a podiatrist indicated they had previously participated, only demographics such as gender, year of birth and year of graduation were shown in addition to new questions. Forced or requested responses were used to minimise missing data, but podiatrists could close and exit the survey at any time. Cookies were used to allow responses to be saved up to 4 h within partial completion.

The following data were collected in waves 2–4 surveys and used in this study:

(i) General demographics: age, gender and years since graduation.
(ii) Overall health and mental health measures: the abbreviated Maslach's Burnout Inventory with an additional three questions relating to job satisfaction domains [16], Brief Resilience Scale [17] and Kessler Psychological Distress Scale (K10) [18].
(iii) Personality, life experiences and risk‐taking behaviours: Ten‐Item Personality Inventory [19], personal life events were collected with the time frame of occurrence and risk taking behaviours, using questions refined through the MABEL survey [20] relating to financial, career (professional) and clinical risk taking.
(iv) Work history: primary work setting, business relationship, number of work locations, hours worked, number of patients seen, consultation times, patient waiting periods, offer bulk billing services, access to leave and professional development.
(v) Job satisfaction: A 10‐item revised job satisfaction scale [21]. An additional 12 questions explored access to taking leave when wanted, taking leave at short notice leave, patient expectations, complex patient cases, peer support, working preferred hours, work schedule predictability, balance between personal and professional commitments, workplace stress and task alignment with qualifications.

### 2.4 Definition of burnout

Burnout was measured using the abbreviated Maslach Burnout Inventory (aMBI) [22, 23]. The aMBI is derived from the original 22‐item Maslach Burnout Inventory human services scale (MBI‐HSS), which was specifically designed to measure burnout among health practitioners. The aMBI has been shown to provide valid and reliable proxies for the MBI‐HSS [24]. The aMBI consists of nine questions across three subscales: emotional exhaustion (three questions); depersonalisation (three questions); and personal accomplishment (three questions). Responses are scored on a seven‐point scale that ranges from 0 (‘never’) to 6 (‘daily’). Three additional questions measuring job satisfaction among health professionals were included [16]. Although the criterion‐related and construct validity of these questions remains unknown, they exhibit face and content validity and have been extensively used to measure job satisfaction in the context of burnout in healthcare [16, 25, 26]. Each of the four subscales were scored by summing their items. Higher scores on the emotional exhaustion and depersonalisation subscales indicated greater levels of burnout in respondents. For emotional exhaustion and depersonalisation, a subscale score of 0–9 was categorised as ‘no to low burnout’ and a score of 10–18 was considered as ‘moderate to high burnout’. In contrast, lower scores on the personal accomplishment and job satisfaction subscales indicated greater levels of burnout in respondents. For personal accomplishment and job satisfaction, a subscale score of 0–9 was categorised as ‘moderate to high burnout’ while a score of 10–18 was considered as ‘no to low burnout’. Participants were classified as experiencing burnout if they scored ‘moderate to high’ in two or more of the four subscales, as per previous studies [5, 27].

### 2.5 Data handling

All data were cleaned to remove participants only completing wave 1, as mental health scales were only included in waves 2–4. Participant responses were also removed if they did not provide complete burnout data or if data were missing for core demographics (gender, age and work location). The most recent responses were retained if participants answered questions in more than one wave. Where participants responded they did not have a change in workplace or living location in their most recent response, job results and living results were aggregated to 1 unique response. A preliminary analysis was conducted to determine if differences existed between data from wave 3 (2019) and wave 4 (2020) relative to participant responses relating to mental health scores, burnout, any impact on job satisfaction or intent to leave the profession. This was due to the timing of data collection in wave 4 during the SARS‐CoV‐2 pandemic and its variable impact around the states and territories in Australia at this timepoint [5]. As no differences were identified the data from waves 2–4, wave 4 data were combined and analysed [5].

Descriptive responses were grouped and recoded whenever possible (e.g. ‘very dissatisfied’ and ‘moderately dissatisfied’ were grouped as ‘dissatisfied’, etc.). Postcode data was recoded using the Modified Monash Model (MMM) to classify locations as either metro areas (MMM 1) or rural areas (MMM 2, 3, 4, 5, 6 and 7) [28]. Variables shown to, or proposed to, have associations with burnout among health practitioners were chosen, including gender, age, years since graduation, mental health measures, personality, life experiences and risk‐taking behaviours, industry career progression intent, workplace factors and job satisfaction.

### 2.6 Statistical analysis

Statistical analysis was performed using the IBM SPSS version 28.0 (IBM Corp., Armonk, NY, USA). Measures were compared between participants classified as experiencing burnout and those classified as not experiencing burnout. Independent sample *t*‐tests were used for continuous data and Chi‐square tests were used for categorical data. Assumptions related to distribution of residuals were checked following *t*‐test analyses. Variables were considered significantly different between groups (burnout vs. no burnout) if *p* < 0.05. Effect sizes for all Chi‐square tests were reported using Phi (φ) for two × two contingency tables and Cramer's V for any table larger than a two × two contingency table. Effect sizes for all *t*‐tests were reported using Cohen's *d*. Three separate multiple logistic regression analyses were conducted to assess the ability of (i) individual characteristics, (ii) workplace variables and (iii) job satisfaction to predict burnout, using a more liberal significance level of *p* < 0.20 to avoid excluding any potential predictor variables. Using separate models recognises individual characteristics, workplace variables and job satisfaction as distinct constructs, allowing us to explore the relative contribution of these three constructs to burnout. The Akaike Information Criterion (AIC) and the Bayesian Information Criterion (BIC) were used to assess the fit of the three models, with the best‐fit model determined by the lowest score. Nagelkerke *R* ^2^ was reported to provide an approximation of the proportion of the variation in the dependent variable (i.e. burnout) that can be explained by the independent variables, with higher values suggesting a better fit of the model to the data.

## 3 RESULTS

A total of 848 participants (15.6% of 5429 registered podiatrists) were included in this study, with 268 participants (31.6% of *n* = 848) identified as experiencing burnout (Table 1).

**TABLE 1:** Comparing the individual characteristics of participants experiencing and not experiencing burnout through univariate analysis (*n* = 848).

Variable	Burnout (*n* = 268)	No burnout (*n* = 580)	*p*‐value	Effect size^a^
Age (years)	37.9 (10.2)	40.0 (10.7)	0.008*^,^ ^b^	0.20
Female, *n* (%)	184 (68.7)	411 (70.9)	0.514	0.02^φ^
Recency, *n* (%)				
0–5 years	120 (44.8)	216 (37.2)	0.111^b^	0.07^V^
6–10 years	50 (18.7)	119 (20.5)	0.111^b^	0.07^V^
>10 years	98 (36.6)	245 (42.2)	0.111^b^	0.07^V^
Additional industry‐based training intended or completed, *n* (%)	45 (16.8)	106 (18.3)	0.599	0.18^φ^
Health, *n* (%)				
Good to excellent	245 (91.4)	558 (96.2)	0.004*	0.099^V^
Poor to fair	23 (8.6)	22 (3.8)	0.004*	0.099^V^
Mental distress^c^, *n* (%)				
Not distressed	164 (61.2)	500 (86.2)	<0.001*^,^ ^b^	0.28^V^
Distressed	104 (38.8)	80 (13.8)	<0.001*^,^ ^b^	0.28^V^
Resilience^d^, *n* (%)				
Low resilience	91 (34.0)	76 (13.1)	<0.001*^,^ ^b^	0.24^V^
Normal to high resilience	177 (66.0)	504 (86.9)	<0.001*^,^ ^b^	0.24^V^
Personality (TIPI)				
Extraversion	5.7 (3.1)	6.3 (3.2)	0.012*^,^ ^b^	0.19
Agreeableness	7.2 (3.0)	7.7 (3.1)	0.053^b^	0.16
Conscientiousness	7.9 (3.2)	8.4 (3.3)	0.039*^,^ ^b^	0.15
Emotional stability	6.5 (3.0)	7.5 (3.2)	<0.001*^,^ ^b^	0.32
Openness to experiences^e^	6.9 (3.1)	7.3 (2.9)	0.044*^,^ ^b^	0.13
Personal life event/s in past 12‐months, *n* (%)	148 (55.2)	295 (50.9)	0.237	0.04^φ^
Financial risk‐taking behaviour (likely), *n* (%)	27 (10.1)	63 (10.9)	0.107^b^	0.07^φ^
Clinical risk‐taking behaviour (likely), *n* (%)	68 (25.4)	153 (26.4)	0.065^b^	0.08^φ^
Career risk‐taking behaviour (likely), *n* (%)	52 (19.4)	100 (17.2)	0.023*	0.09^φ^
aMBI				
Emotional exhaustion	11.8 (3.9)	5.7 (3.6)	<0.001*^,^ ^b^	1.63
Depersonalisation	5.5 (4.7)	2.2 (2.5)	<0.001*^,^ ^b^	0.88
Personal accomplishment	13.0 (3.8)	15.1 (2.5)	<0.001*^,^ ^b^	0.65
Job satisfaction	6.4 (3.5)	7.2 (4.3)	0.015*^,^ ^b^	0.20

*Note*: Values are mean (SD) unless stated.

Abbreviations: aMBI, abbreviated Maslach Burnout Inventory; TIPI, Ten‐Item Personality Inventory.

^a^ Effect sizes reported as Cohen's *d* unless stated (φ = Phi, V = Cramer's V).

^b^ Included in multiple logistic regression analysis as *p* < 0.20.

^c^ Mental distress was determined using the K10 with scores ranging 10–50. Participants classified as not distressed (<22) or distressed (>22).

^d^ Resilience was determined using the Brief Resilience Scale, with scores ranging 0–5. Resilience classified as low (1.00–2.99), normal (3.00–4.30) or high (4.31–5).

^e^ Data transformed prior to analysis.

*p < 0.05.

### 3.1 Differences between participants that did and did not experience burnout

Statistically significant differences (*p* < 0.05) were observed across numerous variables between participants who experienced burnout (*n* = 268) and those who did not (*n* = 580). Participants classified as experiencing burnout were slightly younger, reported poorer health, higher levels of mental distress, scored lower on measures of resilience, extraversion, conscientiousness and emotional stability, were less likely to be open to new experiences and had lower levels of career risk‐taking behaviour. Additionally, they had shorter consultation times, worked more hours, engaged in higher direct patient hours and saw a greater number of patients. They were also more inclined to bulk bill chronic disease management plans and had reduced access to professional development opportunities. Participants experiencing burnout were also more likely to lack a supportive network of peers, not have the ability to take time off or doing so at short notice, experience unrealistic patient expectations and lack support and supervision from a podiatrist with advanced skills. They were also more likely to have unpredictable work hours, experience higher stress from running their own practice, undertake tasks somebody less qualified can do, were less able to work their preferred hours due to work availability and were more likely to want to change their working hours. They also reported lower satisfaction regarding their freedom to choose own method of working, variety of work, physical working conditions, opportunities to use their abilities, recognition of their good work, hours of work, remuneration, amount of responsibility given, how they feel about their job and balance between personal and professional commitments (Tables 1, 2, 3). When the more liberal significance level of *p* < 0.20 was applied, eight additional possible predictors were identified, with participants classified as experiencing burnout being more recent to practice, have lower agreeableness scores, be more likely to have financial risk taking behaviour, more likely to have clinical risk taking behaviour, work in private practice, work across more work locations, more likely to intend to leave the profession within 5 years and the majority of their patients have complex health or social problems (Tables 1, 2, 3).

**TABLE 2:** Comparing the workplace characteristics of participants experiencing and not experiencing burnout through univariate analysis (*n* = 848).

Variable	Burnout (*n* = 268)	No burnout (*n* = 580)	*p*‐value	Effect size^a^
Primary work setting, *n* (%)				
Private practice	179 (66.8)	356 (61.4)	0.106^b^	0.56^V^
Public health service	85 (31.7)	218 (37.6)	0.106^b^	0.56^V^
Business relationship, *n* (%)				
Owner or partner	86 (32.1)	173 (29.8)	0.638	0.06^V^
Salaried	137 (51.1)	297 (51.2)	0.638	0.06^V^
Contractor	33 (12.3)	88 (15.2)	0.638	0.06^V^
Locum	2 (0.7)	7 (1.2)	0.638	0.06^V^
Other	10 (3.7)	15 (2.6)	0.638	0.06^V^
Work location, *n* (%)				
Metro	178 (66.4)	407 (70.2)	0.344	0.03^V^
Rural	86 (32.1)	169 (29.1)	0.344	0.03^V^
Number of colleagues in main workplace				
Podiatrists^c^	4.4 (3.8)	4.4 (3.6)	0.38	0.00
Allied health professionals^c^	37.3 (117.9)	38.5 (132.2)	0.86	0.01
Other health professionals^c^	147.0 (669.1)	135.4 (722.9)	0.87	0.02
Number of work locations	2.5 (2.3)	2.2 (1.9)	0.097^b^	0.14
Work hours per week	27.0 (18.2)	23.8 (17.5)	0.015*^,^ ^b^	0.18
Direct patient hours per week	25.6 (13.4)	23.35 (12.5)	0.018*^,^ ^b^	0.25
Indirect patient hours per week^c^	12.4 (11.7)	11.78 (11.0)	0.654	0.06
Patients per week	45.6 (29.0)	40.6 (28.2)	0.016*^,^ ^b^	0.18
Average consultation time, *n* (%)				
<15 min	14 (5.3)	14 (2.4)	0.023*^,^ ^b^	0.10^V^
16–30 min	202 (76.2)	420 (73.0)	0.023*^,^ ^b^	0.10^V^
>30 min	49 (18.5)	141 (24.5)	0.023*^,^ ^b^	0.10^V^
Average patient waiting period, *n* (%)				
<3 days	105 (39.8)	230 (40.1)	0.988	0.01^V^
4–7 days	73 (27.7)	153 (26.7)	0.988	0.01^V^
7–14 days	45 (17.0)	102 (17.8)	0.988	0.01^V^
>14 days	41 (15.5)	88 (15.4)	0.988	0.01^V^
Bulk bill chronic disease management plans, *n* (%)	105 (41.0)	183 (32.8)	0.023*^,^ ^b^	0.08^φ^
>50% work is home visits/residential aged care, *n* (%)	11 (4.8)	32 (6.7)	0.328	0.04^φ^
Paid annual leave (weeks)^c^	2.6 (3.3)	2.66 (2.9)	0.873	0.03
Unpaid annual leave (weeks)^c^	1.6 (2.6)	1.87 (3.6)	0.615	0.09
Access to paid sick leave, *n* (%)	141 (70.5)	317 (76.4)	0.117^b^	0.06^φ^
Access to professional development, *n* (%)	107 (39.9)	320 (55.2)	<0.001*^,^ ^b^	0.14^φ^
Intending to leave patient care within 5 years, *n* (%)	78 (30.4)	126 (22.5)	0.017*^,^ ^b^	0.08^φ^
Intending to leave podiatry within 5 years, *n* (%)	58 (22.0)	99 (17.3)	0.109^b^	0.06^φ^

*Note*: Values are mean (SD) unless stated.

^a^ Effect sizes reported as Cohen's *d* unless stated (φ = Phi, V = Cramer's V).

^b^ *p* < 0.20 = included in multiple logistic regression analysis.

^c^ Data transformed prior to analysis.

*p < 0.05.

**TABLE 3:** Comparing job satisfaction of participants experiencing and not experiencing burnout through univariate analysis (*n* = 848).

Variable	Burnout (*n* = 268)	No burnout (*n* = 580)	*p*‐value	Effect size^a^
*n* (%) of ‘moderately satisfied’ or 'very satisfied’				
Freedom to choose own method of working	205 (76.5)	517 (89.3)	<0.001*^,^ ^b^	0.17
Amount of variety in work	193 (72.0)	501 (86.4)	<0.001*^,^ ^b^	0.18
Physical working conditions	175 (65.3)	489 (84.5)	<0.001*^,^ ^b^	0.22
Opportunities to use abilities	183 (68.3)	502 (86.7)	<0.001*^,^ ^b^	0.22
Colleagues and fellow workers	192 (77.1)	492 (89.3)	<0.001*^,^ ^b^	0.16
Recognition for good work	150 (56.8)	421 (73.7)	<0.001*^,^ ^b^	0.19
Hours of work	180 (67.2)	477 (82.5)	<0.001*^,^ ^b^	0.17
Remuneration	164 (61.4)	435 (76.2)	<0.001*^,^ ^b^	0.15
Amount of responsibility given	191 (72.9)	508 (89.3)	<0.001*^,^ ^b^	0.21
Feel about job, accounting for all things considered	188 (70.1)	511 (88.1)	<0.001*^,^ ^b^	0.22
*n* (%) of ‘agree’ or 'strongly agree’				
Balance of personal and professional commitments is right	113 (42.2)	394 (67.9)	<0.001*^,^ ^b^	0.25
Poor support network of other similar podiatrists	82 (30.9)	138 (23.9)	0.027*^,^ ^b^	0.09
It is difficult to take time off when wanted	124 (47.0)	171 (29.6)	<0.001*^,^ ^b^	0.18
Can take time off at short notice	133 (52.0)	380 (67.0)	<0.001*^,^ ^b^	0.17
Patients have unrealistic expectations	84 (32.3)	98 (17.3)	<0.001*^,^ ^b^	0.19
Majority of patients have complex health/social problems	172 (65.6)	342 (60.3)	0.095^b^	0.08
Good support/supervision from advanced skilled podiatrists	96 (37.6)	263 (48.0)	<0.001*^,^ ^b^	0.16
Work hours are unpredictable	51 (19.2)	69 (11.9)	0.019*^,^ ^b^	0.10
Running own practice is stressful most of the time	95 (54.0)	91 (23.0)	<0.001*^,^ ^b^	0.32
Often undertake tasks somebody less qualified could do	163 (61.7)	303 (52.7)	0.045*^,^ ^b^	0.09
Cannot work preferred hours due to a lack of available jobs	40 (16.6)	56 (10.8)	0.009*^,^ ^b^	0.11
*n* (%) of ‘yes, would like to decrease hours’				
Would like a change in work hours	69 (28.4)	125 (23.1)	0.101^b^	0.08

*Note*: Values are mean (SD) unless stated.

^a^ Effect sizes reported as Phi.

^b^ *p* < 0.20 = included in multiple logistic regression analysis.

*p < 0.05.

### 3.2 Multiple logistic regression analysis

The multiple logistic regression analysis was statistically significant, *χ* ^2^(17) = 103.4, *p* < 0.001, Nagelkerke *R* ^2^ = 0.161, for *individual characteristics*. The combination of the 13 variables (age, recency of practice, overall health, mental distress, resilience, extraversion, agreeableness, conscientiousness, emotional stability, openness to experiences, financial risk‐taking behaviour, clinical risk‐taking behaviour and career risk‐taking behaviour) approximated 16.1% of the variance in burnout experience and correctly classified 72.4% of cases. The AIC and the BIC of this model was 712.4 and 760.2, respectively. The five subscales of the aMBI were not included in the multiple logistic regression analysis due to multi‐collinearity.

The multiple logistic regression analysis was also statistically significant, *χ* ^2^(10) = 30.4, *p* < 0.001, Nagelkerke *R* ^2^ = 0.068, for *workplace factors.* The combination of the 10 variables (primary work setting, number of work locations, work hours per week, direct patient hours, patients per week, average consultation time, bulk bill chronic disease management plans, access to sick leave, access to professional development opportunities and intending to leave the profession within 5 years) approximated 6.8% of the variance in burnout experience and correctly classified 67.1% of cases. The AIC and the BIC of this model was 712.4 and 760.2, respectively.

The multiple logistic regression analysis was also statistically significant, *χ* ^2^(44) = 92.8, *p* < 0.001, Nagelkerke *R* ^2^ = 0.291, for *measures of work satisfaction.* The combination of the 20 variables relating to job satisfaction approximated 29.1% of the variance in burnout experience and correctly classified 78.8% of cases. The AIC and the BIC of this model was 457.6 and 549.8, respectively.

## 4 DISCUSSION

This study identified that around one third of podiatrists are experiencing burnout and there are multilevel factors associated with it. The evidence was based on a national survey, the PAIGE study comprehensively collecting information about individual characteristics, workplace factors and job satisfaction measures. Differences in these constructs were found between podiatrists that did and did not experience burnout, highlighting the multifaceted nature of burnout and its links to personal and workplace factors. The findings suggest potential areas for targeted interventions to reduce burnout among Australian podiatrists.

Numerous *individual characteristics* were associated with burnout in this study, including younger age, recency of practice, mental distress, resilience and several personality domains. Some individual factors appear unique to the podiatry profession, while others, such as personality [29] and younger age [29, 30], have been shown to be associated with burnout in other healthcare practitioners. Although this study did not explore whether burnout leads to practitioner turnover, it did reveal an association with an intention to leave the profession, aligning with findings in other healthcare fields [31, 32, 33]. Further exploration of this relationship in future podiatry workforce research is warranted, as early career burnout by younger podiatrists may lead to an older workforce distribution [29, 30]. Greater awareness of the risk of burnout by younger podiatrists could guide the implementation of targeted interventions like professional support programs in the workplace along with mindfulness practices and self‐confidence building, which have proven successful among physicians and allied health practitioners [34]. Strategies to reduce burnout may be considered optimal, but all work settings should also prioritise training both workplaces and individuals to effectively recognise signs of burnout. Simultaneously, there should be an emphasis on ensuring podiatrists have adequate access to mental health support and employee assistance programs.

Our study identified several *workplace factors* associated with burnout among podiatrists, supporting previously expressed sentiments that work‐related stress may be the central cause of burnout [1, 29]. Notably, some of these factors, specifically higher workloads, longer hours and seeing more patients, are also observed in other healthcare professions [29, 30]. Strategies aimed at adjusting work patterns and workloads in some health professions have not only reduced burnout among health practitioners but have also enhanced well‐being, work engagement, quality of life, resilience and alleviated stress, anxiety and depression [29, 34]. While these interventions appear valuable, our findings suggest a need for additional strategies for podiatrists with higher workloads and high throughputs, using work supportive rostering practices, scheduled time off and employing into team‐based podiatry where there is more back up and administrative assistance during periods of high workload. Other options could include consolidating the number of work locations, improving working conditions like equipment which can increase efficiency, facilitating professional development opportunities and recognising good work.

Critically, our study identified that low work satisfaction approximated the greatest amount of variance (29.1%) as well as exhibiting the highest prediction accuracy (78.8%) of all the dimensions studied. This indicates that work satisfaction provides the highest level of discrimination between those who do and do not experience burnout and could be applied to screen employee podiatrists during professional reviews. Our finding is consistent with observations among physicians [29, 30]. Like podiatrists in this study, physicians experiencing burnout also face organisational challenges such as limited autonomy and inadequate mentorship [29]. Currently, there is limited research on interventions explicitly targeting job satisfaction in healthcare, its effects on burnout and how to mitigate burnout associated with low job satisfaction. Most interventions primarily focus on increasing individual resilience to work‐related stress or addressing organisational factors and it can be deduced that job satisfaction may improve if the individual and workplace factors already discussed were actioned. Simple actions like acknowledging good work, ensuring satisfactory remuneration and improving physical work conditions could be important.

The study's findings should be considered within the context of both its strengths and limitations. Firstly, this study included 16% of Australian podiatrist respondents, so caution is needed when generalising these results to the entire profession. However, one strength was that the research drew from the largest longitudinal study of Australian podiatrists, with participant demographics closely aligning with the national workforce profile from registration data [15], including meaningful variation in gender, workplace and geographical locations across different outcome and predictor variables. Secondly, as results were obtained from voluntary questionnaires, it is possible that individuals already experiencing burnout, or with a particular interest in the topic, were more likely to participate. The general emphasis of the PAIGE study was not on being a ‘burnout study’ so self‐selection bias is unlikely, though non‐respondents with burnout might have refrained from participating due to their mental health and the length of the survey. Thirdly, our set of predictor variables only approximated between 6.8% and 29.1% of the variance in burnout, which suggests that a large amount of variance remains unaccounted for and may be explained by other variables we did not measure. Furthermore, the prediction accuracy varied between 67.1% and 78.8%, which is only modest and could not be solely relied upon to identify individuals at risk of burnout. Lastly, our study's cross‐sectional nature means it only represents analyses at a point in time.

## 5 CONCLUSION

This study provides a comprehensive analysis of burnout among podiatrists, highlighting differences in various factors between those who experience burnout and those who do not. Burnout is predicted based on individual characteristics, workplace factors and job satisfaction measures among Australian podiatrists. These findings highlight the multifaceted nature of burnout and offer potential avenues for future research and interventions within the podiatry profession. Efforts to reduce burnout among podiatrists can benefit from a holistic approach that considers both personal and workplace factors.

## AUTHOR CONTRIBUTIONS

**Daniel R. Bonanno**: Formal analysis; methodology; resources; writing – original draft; writing – review and editing. **Anna Couch**: Investigation; methodology; project administration; resources; writing – review and editing. **Terry Haines**: Conceptualization; funding acquisition; methodology; writing – review and editing. **Hylton B. Menz**: Conceptualization; funding acquisition; formal analysis; methodology; writing – review and editing. **Belinda G. O'Sullivan**: Conceptualization; methodology; writing – review and editing. **Cylie M. Williams**: Conceptualization; funding acquisition; formal analysis; investigation; methodology; project administration; resources; writing – review and editing.

## CONFLICT OF INTEREST STATEMENT

CMW is an Associate Editor of the *Journal of Foot and Ankle Research.* It is journal policy to remove editors from the peer review processes for papers they have co‐authored. All other authors declare that they have no competing interests.

## ETHICS STATEMENT

Approval was provided by the Monash University Human Research Ethics Committee (19959).

## CONSENT FOR PUBLICATION

Not applicable.

## Data Availability

Data sharing is not applicable to this article as no new data were created or analysed in this study.
